# 54 A Retrospective Review of Copper and Zinc Levels in Burn Injured Patients

**DOI:** 10.1093/jbcr/irae036.046

**Published:** 2024-04-17

**Authors:** Billy Jay Taylor, Claudia Islas, Curt C Bay, Asia N Quan, Karen J Richey, Kevin N Foster

**Affiliations:** Valleywise Health, Phoenix, AZ; Arizona Burn Center/Valleywise Health Medical Center, Phoenix, AZ; A.T. Still University, Mesa, AZ; Arizona Burn Center Valleywise Health, Phoenix, AZ; Valleywise Health, Phoenix, AZ; Arizona Burn Center/Valleywise Health Medical Center, Phoenix, AZ; A.T. Still University, Mesa, AZ; Arizona Burn Center Valleywise Health, Phoenix, AZ; Valleywise Health, Phoenix, AZ; Arizona Burn Center/Valleywise Health Medical Center, Phoenix, AZ; A.T. Still University, Mesa, AZ; Arizona Burn Center Valleywise Health, Phoenix, AZ; Valleywise Health, Phoenix, AZ; Arizona Burn Center/Valleywise Health Medical Center, Phoenix, AZ; A.T. Still University, Mesa, AZ; Arizona Burn Center Valleywise Health, Phoenix, AZ; Valleywise Health, Phoenix, AZ; Arizona Burn Center/Valleywise Health Medical Center, Phoenix, AZ; A.T. Still University, Mesa, AZ; Arizona Burn Center Valleywise Health, Phoenix, AZ; Valleywise Health, Phoenix, AZ; Arizona Burn Center/Valleywise Health Medical Center, Phoenix, AZ; A.T. Still University, Mesa, AZ; Arizona Burn Center Valleywise Health, Phoenix, AZ

## Abstract

**Introduction:**

Micronutrient deficiencies are common in critically ill patients. Both zinc (Zn) and copper (Cu) are important for wound healing and are commonly deficient in severely burned patients. Zn and Cu compete for the same absorption pathways and the simultaneous oral supplementation of both may severely hinder the body’s ability to absorb Cu. The purpose of this study was to better understand serum Zn and Cu levels in patients with a burn injury of ≥ 20% TBSA and the effect of Zn replacement on Cu levels.

**Methods:**

This was a retrospective chart review of adult patients admitted over a 3-year period who had copper and zinc levels drawn as part of their routine and usual care. Descriptive statistics were calculated for demographic and clinical characteristic Spearman’s rank correlation was computed to assess the relationship between Zinc and Copper levels.

**Results:**

A total of 85 patients met inclusion/exclusion criteria. Mean age was 44 years, mean total body surface area (TBSA) was 36%, mean BMI was 28.3. The population was predominantly male 77% (n=65), mean length of stay 47.7 days, mean ICU days 36, mean ventilator days 36. Mortality for the group was 26% (n=21). There was a positive correlation between Cu and Zn for the first two lab draws (p < .01). At the time of the third serum level, this correlation was diminished, and at tests 4 and 5 there was a negative correlation between Cu and Zn, that did not reach significance. When copper levels were correlated over time, there was a positive correlation between the first two levels (p < .01), levels 3 and 4 (p < .01) and levels 4 and 5 (p < .01). Beyond level 5 statistical power was inadequate to support hypothesis testing.

**Conclusions:**

Initially, copper and zinc were positively correlated but over time, this relationship reversed and became negative. This may suggest a shift in the receptor binding competition between the two micronutrients secondary to zinc supplementation beyond that provided by enteral and oral nutrition. Larger scale research is warranted to ensure statistical power and better understand these relationships.

**Applicability of Research to Practice:**

Zinc and copper levels should continue to be monitored until evidence-based protocols can be developed to further guide testing.

